# Self-reported dark chocolate consumption and depressive symptoms among community-dwelling older adults in Nagoya, Japan: a cross-sectional study

**DOI:** 10.3389/fnut.2026.1924057

**Published:** 2026-09-11

**Authors:** Shinnosuke Nosaka, Sho Nakakubo, Kanon Abe, Yuka Misu, Yuto Kiuchi, Kota Tsutsumimoto, Kentaro Nakamura, Midori Natsume, Mayuki Sasaki, Hiroyuki Shimada, Takao Suzuki

**Affiliations:** 1 Department of Preventive Gerontology, Center for Gerontology and Social Science, Research Institute, National Center for Geriatrics and Gerontology, Obu, Japan; 2 Digital Health Research Team, Center for Gerontology and Social Science, Research Institute, National Center for Geriatrics and Gerontology, Obu, Japan; 3 Department of Occupational Therapy, Faculty of Rehabilitation, Kobe Gakuin University, Kobe, Japan; 4 Health Science Research Unit, R&D Division, Meiji Co., Ltd., Hachioji, Japan; 5 Cognitive Function Research, Aging Research (Partnership Field), Nagoya University Graduate School of Medicine, Nagoya, Japan; 6 Medical Science Division, Department of Medical Sciences, Graduate School of Medicine, Science and Technology, Shinshu University, Matsumoto, Japan

**Keywords:** cocoa, cognitive function, dark chocolate, depressive symptoms, Japan, older adults

## Abstract

**Introduction:**

Evidence regarding the association between dark chocolate consumption and depressive symptoms remains limited, particularly among older adults.

**Objective:**

To examine the association between self-reported dark chocolate consumption and depressive symptoms among community-dwelling older adults in Nagoya, Japan.

**Methods:**

This cross-sectional study included 3,910 adults aged ≥ 70 years, of whom 2,957 were analyzed. Depressive symptoms were assessed using the Japanese version of the 15-item Geriatric Depression Scale (GDS-15-J). Dark chocolate consumption and depressive symptoms were assessed at the same study time point. Participants were classified into four groups: rare or no chocolate consumption, chocolate consumption without dark chocolate, dark chocolate consumption once or twice per week or more for ≤2 years, and dark chocolate consumption once or twice per week or more for >2 years. Logistic regression estimated odds ratios (ORs) and 95% confidence intervals (CIs) after adjustment of demographic, socioeconomic, lifestyle, and clinical covariates. Sensitivity analyses assessed robustness.

**Results:**

Among the 2,957 participants, 48.4% were women, and the mean age was 78.0 ± 5.0 years. Dark chocolate consumption for >2 years was reported by 24.6%. In the fully adjusted model, participants reporting dark chocolate consumption for >2 years had lower estimated odds of depressive symptoms than those who reported rare or no chocolate consumption, although the confidence interval included the null (OR, 0.73; 95% CI, 0.53–1.01; *p* = 0.055). Multiple imputation yielded a similar estimate (OR, 0.69; 95% CI, 0.51–0.94; *p* = 0.020). Analyses restricted to participants with Mini-Mental State Examination (MMSE) scores ≥ 27 and ≥28 also yielded estimates in the same direction (OR, 0.66; 95% CI, 0.43–1.00; *p* = 0.051 and OR, 0.68; 95% CI, 0.42–1.12; *p* = 0.132, respectively). Among chocolate consumers, dark chocolate consumption for >2 years was associated with lower odds of depressive symptoms than chocolate consumption without dark chocolate (OR, 0.62; 95% CI, 0.43–0.88; *p* = 0.008).

**Conclusion:**

Self-reported dark chocolate consumption for >2 years may be associated with lower odds of depressive symptoms among community-dwelling older adults in Nagoya, Japan. Prospective and interventional studies are needed to clarify the temporal and causal nature of this association.

## 1 Introduction

Chocolate has been investigated for its potential health-promoting properties, particularly in relation to cardiovascular health. Its consumption may have beneficial effects on key cardiovascular risk markers, including blood pressure, serum lipid profiles, and endothelial function (1, 2). These effects have been attributed to cocoa polyphenols, particularly flavanols such as procyanidins and epicatechin, through their antioxidant activity and enhancement of nitric oxide synthesis (3, 4). Consequently, dark chocolate is widely considered to have greater potential health benefits because of its higher cocoa polyphenol content. Consistent with this view, a recent study reported the benefits of dark chocolate compared with milk chocolate (5). Furthermore, cocoa polyphenols and polyphenol-rich dark chocolate may improve cognitive function. For instance, epicatechin has been shown to upregulate the expression of neurogenesis markers and improve short-term memory *in vivo* (6). Clinical studies have reported similar findings. Natsume et al. reported increased brain-derived neurotrophic factor (BDNF) levels following the consumption of 25 g/day of dark chocolate containing 72% cocoa for 4 weeks (7), whereas Lamport et al. reported improved episodic memory 2 h after the consumption of 35 g of dark chocolate containing 70% cocoa (8).

The potential effects of dark chocolate may extend beyond cognitive function to psychological well-being, as cocoa polyphenol consumption may enhance feelings of calmness and contentment (7). However, evidence regarding the association between dark chocolate consumption and depressive symptoms remains limited. Although some epidemiological studies (9) and intervention studies involving dark chocolate, cocoa extracts, or polyphenol-rich diets (10–12) have reported beneficial associations or effects, most have focused on the general adult population, leaving limited evidence in older adults. Depression is common among older adults (13) who may be particularly vulnerable to associated risk factors, including physical illness, disability, sleep disturbances, social loss, and cognitive decline (14, 15). This issue is particularly relevant in Japan, where adults aged ≥ 70 years account for approximately 23% of the total population (16). Identifying potentially modifiable dietary factors associated with depressive symptoms in this large and growing population may therefore have clinical and public health relevance. Nevertheless, evidence regarding the association between dark chocolate consumption and depressive symptoms among older Japanese adults remains limited.

In the present study, we therefore aimed to examine the association between self-reported dark chocolate consumption and depressive symptoms among community-dwelling older adults in Nagoya, Japan.

## 2 Materials and methods

### 2.1 Design

This cross-sectional analysis used data from the National Center for Geriatrics and Gerontology–Study of Geriatric Syndromes (NCGG–SGS) (17), a population-based, observational, prospective cohort study. The NCGG–SGS aims to establish a screening system for geriatric syndromes and to test evidence-based interventions designed to prevent geriatric conditions (18). This study was reported in accordance with the Strengthening the Reporting of Observational Studies in Epidemiology (STROBE) statement.

### 2.2 Participants

Participants were recruited from residents of Midori Ward, Nagoya City, Japan, using the resident registry as the sampling frame and a census-based invitation approach. A total of 35,108 residents were invited by mail to participate in the study. Of these, 3,910 community-dwelling older adults underwent the study assessment between June 20, 2023, and August 22, 2024, corresponding to a participation rate of 11.1%. The inclusion criteria were residence in Midori Ward, Nagoya City, at the time of the survey and age ≥ 70 years.

**FIGURE 1:**
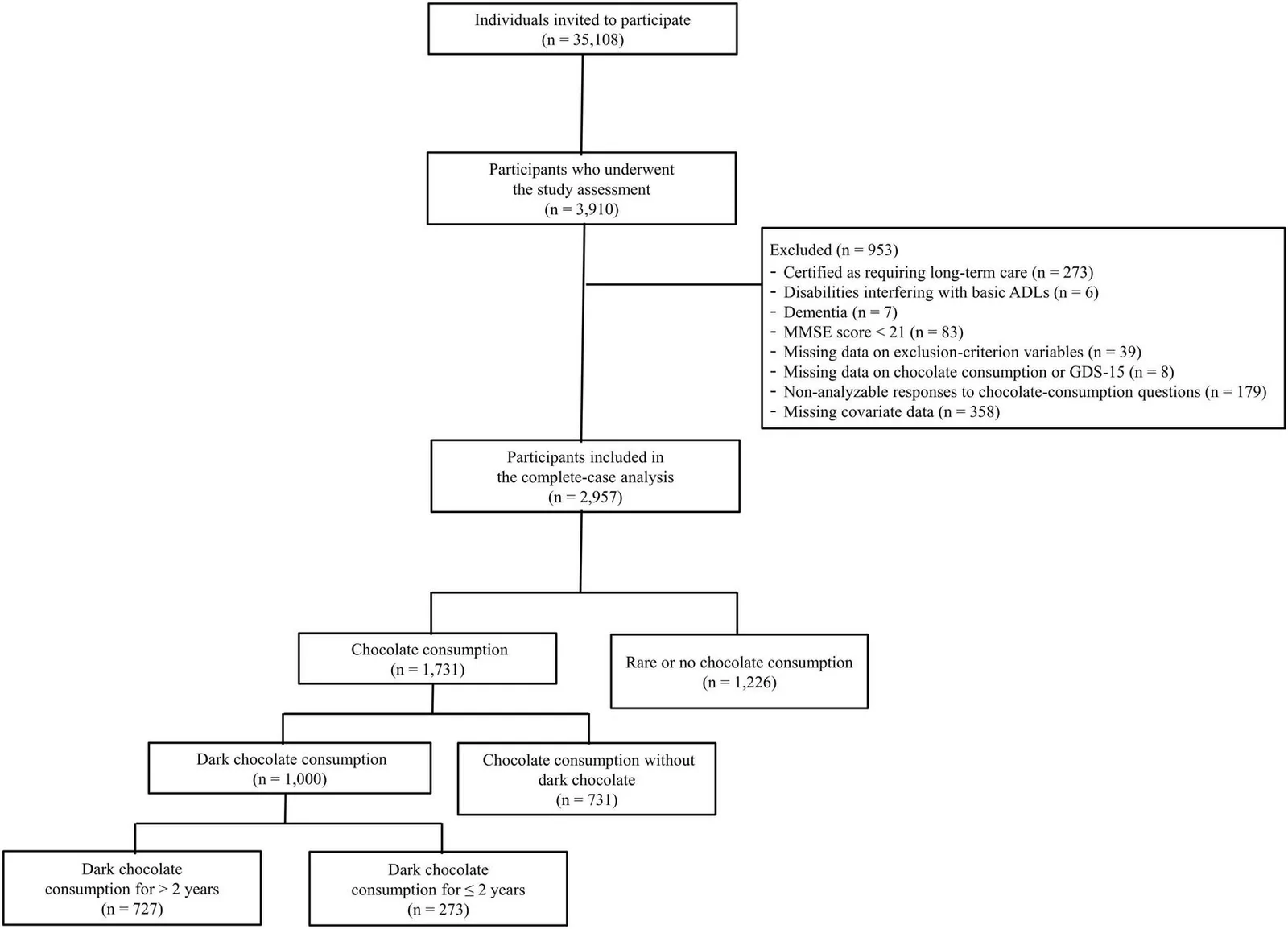
Flowchart of participant selection and classification according to chocolate and dark chocolate consumption. Exclusions were applied sequentially in the order shown. Each participant was counted only once according to the first applicable exclusion criterion; therefore, the exclusion categories were mutually exclusive. Non-analyzable responses referred to “unable to answer” responses to the chocolate consumption questions.

Exclusion criteria were applied sequentially and were mutually exclusive. Participants were excluded if they were certified as requiring long-term care under the Japanese public long-term care insurance system (*n* = 273), had disabilities interfering with basic activities of daily living (*n* = 6), had dementia (*n* = 7), had an MMSE score < 21 (*n* = 83), or had missing data on variables required to determine the exclusion criteria (*n* = 39). Participants with an MMSE score < 21 were excluded to reduce the potential influence of cognitive impairment on the reliability of responses to the self-administered questionnaire. This cutoff has also been used to exclude participants with suspected moderate dementia in previous NCGG-SGS studies (18, 19). Participants were further excluded because of missing data on chocolate consumption or the Japanese version of the 15-item Geriatric Depression Scale (GDS-15-J) (*n* = 8), non-analyzable responses to the chocolate-consumption questions (*n* = 179), or missing covariate data (*n* = 358). No additional exclusion criteria beyond those described above were applied. Ultimately, 2,957 participants were included in the complete-case analysis (Figure 1).

### 2.3 Ethical considerations

All participants provided written informed consent before participation. The study protocol was approved by the Ethics Committee of the National Center for Geriatrics and Gerontology (approval no. 1440). This study was conducted in accordance with the principles of the Declaration of Helsinki (20).

### 2.4 Classification of dark chocolate consumption

The chocolate and cocoa consumption questions were developed by the research group for the present survey. Similar response categories for assessing food-consumption frequency were used in a previous study by our group (21), and a subsequent study classified participants who consumed cheese at least once per week as cheese consumers (22). However, the chocolate and cocoa consumption questions used in the present study have not been formally validated, and their reproducibility has not been evaluated.

Participants completed a self-administered questionnaire assessing the frequency, type, and duration of habitual chocolate consumption. They reported the frequency of chocolate consumption during approximately the past week using the following response categories: “almost every day,” “once every 2 days,” “once or twice per week,” “hardly ever,” and “unable to answer/do not know.” Dark chocolate was defined as chocolate containing ≥70% cocoa (23). The frequency of dark chocolate consumption and the duration of continuous consumption at the reported frequency were assessed separately. Participants who reported consuming dark chocolate in the “once or twice per week” category or more frequently were subsequently asked to report the duration for which they had continuously consumed dark chocolate at that frequency, using the following categories: “within 6 months,” “6 months to 2 years,” “2–5 years,” “5 years or more,” and “unable to answer/do not know.”

Based on these responses, participants were classified into four groups: rare or no chocolate consumption, chocolate consumption without dark chocolate, dark chocolate consumption for ≤2 years, and dark chocolate consumption for >2 years. Participants who reported “hardly ever” for overall chocolate consumption were classified as having rare or no chocolate consumption. Participants who consumed chocolate but reported “hardly ever” for dark chocolate consumption were classified as consuming chocolate without dark chocolate. Participants who reported dark chocolate consumption once or twice per week or more were classified according to the reported duration of continuous consumption as either ≤2 years or >2 years. The 2-years cutoff was based on the prespecified duration categories in the questionnaire rather than on an established clinical threshold; for the primary analysis, the original four duration categories were combined into ≤2 years and >2 years. The original four duration categories were additionally examined in a sensitivity analysis.

The questionnaire assessed self-reported frequency and duration of consumption but did not assess the amount of chocolate consumed. The original Japanese questionnaire, English translation, and response options are provided in the Supplementary Material.

### 2.5 Definition of depressive symptoms

Depressive symptoms were assessed using the GDS-15-J (24, 25). In the primary analysis, depressive symptoms were defined as a GDS-15-J score ≥ 6. The 5/6 cutoff was selected for consistency with previous NCGG-SGS studies (26, 27).

### 2.6 Other covariates

Potential confounders were selected based on their demographic, socioeconomic, lifestyle, dietary, and health-related relevance to the association between dark chocolate consumption and depressive symptoms. The covariates included age, sex, years of education, equivalent income, body mass index (BMI), current smoking, current alcohol consumption, Dietary Variety Score (DVS), diabetes mellitus, hyperlipidemia, hypertension, heart disease, and MMSE score. Information on chronic diseases was obtained through nurse-administered interviews conducted on the day of the assessment and, when necessary, confirmed using the participants’ medication notebooks. Antidepressant medication use, assessed for descriptive and sensitivity analyses, was ascertained in the same manner. These data were not obtained directly from medical records. Other questionnaire items and assessments were administered by research staff trained in the standardized assessment procedures used in our research department.

Equivalent income was calculated by dividing household income by the square root of household size and was categorized as <2.0 million yen, 2.0–3.99 million yen, and ≥4.0 million yen (28, 29). BMI was calculated as body weight in kilograms divided by height in meters squared. Dietary variety was assessed using the DVS, based on the daily consumption of 10 food groups: fish and shellfish, meat, eggs, milk, soybean products, green and yellow vegetables, seaweed, potatoes, fruit, and fats and oils (30). Each food group consumed daily contributed 1 point, yielding a total score ranging from 0 to 10, with higher scores indicating greater dietary variety. Cognitive function was assessed using the MMSE, which evaluates orientation, registration, attention, calculation, recall, and language. The total score ranges from 0 to 30, with higher scores indicating better cognitive function (31, 32).

### 2.7 Statistical analyses

Analyses were based on the four chocolate-consumption groups defined above: rare or no chocolate consumption, chocolate consumption without dark chocolate, dark chocolate consumption for ≤2 years, and dark chocolate consumption for >2 years. The distributions of continuous variables were assessed using histograms, Q–Q plots, and tests of normality. Because formal normality tests are sensitive to minor deviations from normality in large samples, distributional shape was also considered. Continuous variables are presented as means ± standard deviations, and categorical variables as numbers and percentages. Differences among the four groups were examined using one-way analysis of variance for continuous variables and Pearson’s chi-squared test for categorical variables. Fisher’s exact test was used for antidepressant medication use because of small expected cell counts.

In the primary analysis, logistic regression was used to estimate odds ratios (ORs) and 95% confidence intervals (CIs), with depressive symptoms (GDS-15-J score ≥ 6) as the outcome and chocolate consumption status as the exposure. The rare or no chocolate consumption group served as the reference. The adjusted model included age, sex, years of education, equivalent income, BMI, current smoking, current alcohol consumption, DVS, diabetes mellitus, hyperlipidemia, hypertension, heart disease, and MMSE score as covariates.

A direct comparison of dark chocolate consumption for >2 years versus ≤2 years was performed in the fully adjusted model. An omnibus Wald test was also performed to assess the overall association between the four-category chocolate consumption variable and depressive symptoms.

To distinguish the association with dark chocolate from that with chocolate consumption in general, an additional analysis was restricted to chocolate consumers and used a three-group classification: chocolate consumption without dark chocolate, dark chocolate consumption for ≤2 years, and dark chocolate consumption for >2 years. The chocolate consumption without dark chocolate group served as a reference, and the same covariates as in the primary analysis were included.

Several sensitivity analyses were conducted to assess the robustness of the findings. First, the original four duration categories of dark chocolate consumption (“within 6 months,” “6 months to 2 years,” “2–5 years,” “5 years or more”) were examined separately, and a Wald test was used to assess heterogeneity across the duration categories. Second, because a validation study in Japanese older adults reported a 6/7 cutoff (GDS-15-J ≥ 7) as the optimal screening threshold (25), the fully adjusted logistic regression analysis was repeated using GDS-15-J ≥ 7 to define depressive symptoms. Third, to assess the robustness of the findings to dichotomization of the GDS-15-J score, the total score was analyzed as the outcome. Given the observed distribution of GDS-15-J scores, negative binomial regression with a log link was used to estimate rate ratios (RRs) and 95% CIs. Fourth, the fully adjusted analysis was repeated after excluding participants using antidepressant medications. Fifth, modified Poisson regression with robust variance estimation was performed to estimate adjusted prevalence ratios (PRs) and 95% CIs.

In addition, *post hoc* sensitivity analyses restricted to participants with MMSE scores ≥ 27 and ≥28 were conducted to examine the robustness of the findings among participants with relatively preserved cognitive function (33).

To assess the potential impact of missing data, multiple imputation by chained equations was performed as a sensitivity analysis to the complete-case analysis, using the mice package in R, to generate 50 imputed datasets. Predictive mean matching was used to impute missing BMI values, and polytomous logistic regression was used to impute missing equivalent-income values. Estimates from the imputed datasets were pooled using Rubin’s rules.

Linearity of the logit for continuous covariates was assessed using restricted cubic splines for age, years of education, BMI, DVS, and MMSE score. Multicollinearity was assessed using variance inflation factors (VIFs) and tolerance values. Model fit and calibration were assessed using the Hosmer–Lemeshow goodness-of-fit test, −2 log likelihood, Cox–Snell R^2^, and Nagelkerke R^2^. Influential observations were assessed using Cook’s distance, leverage values, and standardized residuals. In addition, a diagnostic reanalysis excluding observations with Cook’s distance > 0.10 was performed to assess whether relatively influential observations materially altered the primary association estimate. This threshold was used for diagnostic purposes rather than as a prespecified criterion for excluding observations.

No *a priori* sample-size calculation was performed specifically for the present analysis because the sample size was determined by the number of eligible participants available from the existing cohort rather than by this specific analysis. No separate prespecified statistical analysis plan was developed specifically for the present analysis of dark chocolate consumption and depressive symptoms. All statistical tests were two-sided, and *p*-values < 0.05 were considered statistically significant. Statistical analyses were performed using IBM SPSS Statistics version 29.0 (IBM Japan, Tokyo, Japan) and R version 4.4.0 (R Foundation for Statistical Computing, Vienna, Austria).

## 3 Results

A total of 2,957 participants were included in the complete-case analysis. The mean age was 78.0 ± 5.0 years, and 1,432 (48.4%) participants were women. Overall, 1,731 (58.5%) participants reported chocolate consumption, whereas 1,226 (41.5%) reported rare or no chocolate consumption. Among participants who reported chocolate consumption, 1,000 reported consuming dark chocolate once or twice per week or more frequently, and 731 consumed chocolate without dark chocolate. Among the 1,000 participants who consumed dark chocolate, 273 had consumed it for ≤2 years and 727 for >2 years, corresponding to 9.2% and 24.6% of the total study population, respectively (Figure 1).

Participant characteristics according to the four chocolate consumption categories are shown in Table 1. The prevalence of depressive symptoms ranged from 8.3% to 13.2% across the four groups and was lowest among participants who reported dark chocolate consumption for >2 years.

**TABLE 1:** Participant characteristics.

Characteristic	Overall	Rare or no chocolate consumption	Chocolate consumption without dark chocolate	Dark chocolate consumption for ≤2 years	Dark chocolate consumption for >2 years	*P*
	(*n* = 2,957)	(*n* = 1,226)	(*n* = 731)	(*n* = 273)	(*n* = 727)	
Age, years	78.0 ± 5.0	78.2 ± 5.1	77.7 ± 5.2	77.8 ± 4.7	77.9 ± 4.8	0.151
Women, *n* (%)	1,432 (48.4)	519 (42.3)	358 (49.0)	158 (57.9)	397 (54.6)	<0.001
Years of education	13.1 ± 2.5	12.9 ± 2.6	13.2 ± 2.6	13.1 ± 2.5	13.2 ± 2.5	0.034
Equivalent income, n (%)						
Low (<2.00 million yen)	1,310 (44.3)	541 (44.1)	340 (46.5)	121 (44.3)	308 (42.4)	0.744
Middle (2.00–3.99 million yen)	1,303 (44.1)	540 (44.0)	315 (43.1)	117 (42.9)	331 (45.5)	
High (≥4.00 million yen)	344 (11.6)	145 (11.8)	76 (10.4)	35 (12.8)	88 (12.1)	
Body mass index, kg/m^2^	22.9 ± 3.1	23.0 ± 3.1	23.0 ± 3.1	22.8 ± 3.2	22.8 ± 3.0	0.638
Current smoking, *n* (%)	142 (4.8)	71 (5.8)	33 (4.5)	8 (2.9)	30 (4.1)	0.134
Current alcohol consumption, *n* (%)	1,346 (45.5)	586 (47.8)	329 (45.0)	115 (42.1)	316 (43.5)	0.161
Dietary Variety Score, points	4.9 ± 2.2	4.6 ± 2.2	4.8 ± 2.2	5.0 ± 2.0	5.4 ± 2.1	<0.001
Diabetes mellitus, *n* (%)	449 (15.2)	216 (17.6)	98 (13.4)	41 (15.0)	94 (12.9)	0.016
Hyperlipidemia, *n* (%)	1,094 (37.0)	437 (35.6)	298 (40.8)	93 (34.1)	266 (36.6)	0.091
Hypertension, *n* (%)	1,478 (50.0)	639 (52.1)	359 (49.1)	125 (45.8)	355 (48.8)	0.189
Heart disease, *n* (%)	556 (18.8)	241 (19.7)	108 (14.8)	61 (22.3)	146 (20.1)	0.010
MMSE score, points	26.8 ± 2.3	26.7 ± 2.3	27.0 ± 2.3	26.9 ± 2.2	27.0 ± 2.3	0.002
Medication for depression, *n* (%)	19 (0.6)	11 (0.9)	3 (0.4)	3 (1.1)	2 (0.3)	0.215
Depressive symptoms, *n* (%)	328 (11.1)	143 (11.7)	89 (12.2)	36 (13.2)	60 (8.3)	0.038

Values are presented as mean ± standard deviation or number (percentage), as appropriate. Continuous variables were compared across the four chocolate consumption groups using one-way analysis of variance, and categorical variables were compared using Pearson’s chi-squared test, except for antidepressant medication use, which was compared using Fisher’s exact test because of small expected cell counts. Two-sided *p*-values < 0.05 were considered statistically significant. BMI, body mass index; MMSE, Mini-Mental State Examination.

The results of the logistic regression analysis examining the association between chocolate consumption status and depressive symptoms are presented in Table 2. In the crude model, compared with rare or no chocolate consumption, the OR for depressive symptoms was lower among participants reporting dark chocolate consumption for >2 years (OR = 0.68, 95% CI = 0.50–0.94; *p* = 0.017). The estimate was similar after adjustment for age and sex (Model 1: OR = 0.68, 95% CI = 0.50–0.94; *p* = 0.019). In the fully adjusted model (Model 2), the OR for dark chocolate consumption for >2 years was 0.73 (95% CI = 0.53–1.01; *p* = 0.055). The corresponding estimates were OR = 1.10 (95% CI = 0.83–1.47; *p* = 0.509) for chocolate consumption without dark chocolate and OR = 1.18 (95% CI = 0.79–1.76; *p* = 0.426) for dark chocolate consumption for ≤2 years. A direct comparison of dark chocolate consumption for >2 years versus ≤2 years yielded an adjusted OR of 0.618 (95% CI = 0.396–0.965; *p* = 0.034). The omnibus Wald test for the four-category chocolate consumption variable yielded χ^2^ = 6.837 (df = 3; *p* = 0.077) in Model 2.

**TABLE 2:** Odds ratios (ORs) and 95% confidence intervals (CIs) for depressive symptoms across four groups based on chocolate consumption status.

Variable	Depressive symptoms	Crude model		Model 1		Model 2	
	*n* (%)	OR (95% CI)	*P*	OR (95% CI)	*P*	OR (95% CI)	*P*
Rare or no chocolate consumption (*n* = 1,226)	143 (11.7)	Ref.		Ref.		Ref.	
Chocolate consumption without dark chocolate (*n* = 731)	89 (12.2)	1.05 (0.79–1.40)	0.735	1.06 (0.80–1.40)	0.710	1.10 (0.83–1.47)	0.509
Dark chocolate consumption for ≤2 years (*n* = 273)	36 (13.2)	1.15 (0.78–1.70)	0.483	1.15 (0.78–1.71)	0.476	1.18 (0.79–1.76)	0.426
Dark chocolate consumption for >2 years (*n* = 727)	60 (8.3)	0.68 (0.50–0.94)	0.017	0.68 (0.50–0.94)	0.019	0.73 (0.53–1.01)	0.055
Overall *p*-value for chocolate consumption			0.040		0.039		0.077
Age, per year				1.01 (0.99–1.04)	0.251	1.01 (0.99–1.04)	0.381
Sex (reference: women)				0.98 (0.78–1.24)	0.867	0.85 (0.64–1.12)	0.253
Years of education, per year						1.00 (0.95–1.06)	0.870
Equivalent income							
Low (<2.00 million yen)						Ref.	
Middle (2.00–3.99 million yen)						0.83 (0.65–1.06)	0.143
High (≥4.00 million yen)						0.28 (0.16–0.50)	<0.001
Body mass index, per kg/m^2^						0.97 (0.93–1.01)	0.156
Current smoking (reference: no)						1.98 (1.25–3.15)	0.004
Current alcohol consumption (reference: no)						0.93 (0.72–1.20)	0.595
Dietary Variety Score, per point						0.93 (0.88–0.98)	0.013
Diabetes mellitus (reference: no)						1.29 (0.94–1.76)	0.109
Hyperlipidemia (reference: no)						0.89 (0.69–1.15)	0.360
Hypertension (reference: no)						0.98 (0.76–1.25)	0.867
Heart disease (reference: no)						1.75 (1.33–2.31)	<0.001
MMSE score, per point						0.99 (0.94–1.04)	0.627

Model 1 was adjusted for age and sex. Model 2 was adjusted for age, sex, years of education, equivalent income, body mass index (BMI), smoking status, alcohol consumption, Dietary Variety Score (DVS), diabetes mellitus, hyperlipidemia, hypertension, heart disease, and Mini-Mental State Examination (MMSE) score. Depressive symptoms were defined as a 15-item Geriatric Depression Scale (GDS-15-J) score ≥ 6. Two-sided *p*-values < 0.05 were considered statistically significant. In Model 2, the direct comparison of dark chocolate consumption for >2 years versus ≤2 years yielded an adjusted OR of 0.618 (95% CI, 0.396–0.965; *p* = 0.034). The omnibus Wald test for the four-category chocolate consumption variable in Model 2 yielded χ^2^ = 6.837 (df = 3; *p* = 0.077). ORs and 95% CIs were estimated using logistic regression.

Among chocolate consumers, using chocolate consumption without dark chocolate as the reference category, dark chocolate consumption for >2 years was associated with lower odds of depressive symptoms in the fully adjusted model (OR = 0.62, 95% CI = 0.43–0.88; *p* = 0.008). The corresponding estimate for dark chocolate consumption for ≤2 years was OR = 1.00 (95% CI = 0.66–1.54; *p* = 0.984). The omnibus Wald test for the three-category dark chocolate consumption variable yielded χ^2^ = 8.115 (df = 2; *p* = 0.017) (Table 3).

**TABLE 3:** Odds ratios (ORs) and 95% confidence intervals (CIs) for depressive symptoms across three groups based on dark chocolate consumption status.

Variable	Depressive symptoms	Crude model		Model 1		Model 2	
	*n* (%)	OR (95% CI)	*P*	OR (95% CI)	*P*	OR (95% CI)	*P*
Chocolate consumption without dark chocolate (*n* = 731)	89 (12.2)	Ref.		Ref.		Ref.	
Dark chocolate consumption for ≤2 years (*n* = 273)	36 (13.2)	1.10 (0.72–1.66)	0.666	1.07 (0.71–1.63)	0.738	1.00 (0.66–1.54)	0.984
Dark chocolate consumption for >2 years (*n* = 727)	60 (8.3)	0.65 (0.46–0.92)	0.014	0.64 (0.45–0.90)	0.011	0.62 (0.43–0.88)	0.008
Overall *p*-value for dark chocolate consumption			0.019		0.017		0.017
Age, per year				1.04 (1.01–1.07)	0.022	1.03 (1.00–1.06)	0.066
Sex (reference: women)				0.79 (0.58–1.08)	0.133	0.64 (0.44–0.94)	0.022
Years of education, per year						1.04 (0.97–1.11)	0.297
Equivalent income							
Low (<2.00 million yen)						Ref.	
Middle (2.00–3.99 million yen)						0.88 (0.63–1.22)	0.435
High (≥4.00 million yen)						0.31 (0.14–0.65)	0.002
Body mass index, per kg/m^2^						0.99 (0.94–1.04)	0.653
Current smoking (reference: no)						1.86 (0.89–3.85)	0.097
Current alcohol consumption (reference: no)						0.91 (0.65–1.29)	0.604
Dietary Variety Score, per point						0.98 (0.90–1.05)	0.515
Diabetes mellitus (reference: no)						1.20 (0.77–1.89)	0.416
Hyperlipidemia (reference: no)						0.71 (0.51–1.01)	0.055
Hypertension (reference: no)						0.96 (0.69–1.33)	0.805
Heart disease (reference: no)						1.85 (1.27–2.69)	0.001
MMSE score, per point						0.97 (0.90–1.04)	0.388

Model 1 was adjusted for age and sex. Model 2 was adjusted for age, sex, years of education, equivalent income, body mass index (BMI), smoking status, alcohol consumption, Dietary Variety Score (DVS), diabetes mellitus, hyperlipidemia, hypertension, heart disease, and Mini-Mental State Examination (MMSE) score. Depressive symptoms were defined as a 15-item Geriatric Depression Scale (GDS-15-J) score ≥ 6. Participants were classified into three groups according to dark chocolate consumption status: chocolate consumption without dark chocolate, dark chocolate consumption for ≤2 years, and dark chocolate consumption for >2 years. Chocolate consumption without dark chocolate was used as the reference category. Two-sided *p*-values < 0.05 were considered statistically significant. The omnibus Wald test for the three-category dark chocolate consumption variable in Model 2 yielded χ^2^ = 8.115 (df = 2; *p* = 0.017). ORs and 95% CIs were estimated using logistic regression.

The cross-tabulation of self-reported dark chocolate consumption frequency and retrospectively reported consumption duration among the 1,000 participants who consumed dark chocolate once or twice per week or more frequently is presented in Supplementary Table 1. In the sensitivity analysis using the original four duration categories, with consumption “within 6 months” as the reference, the adjusted ORs for depressive symptoms were 0.947 (95% CI = 0.458–1.957; *p* = 0.883) for “6 months to 2 years”, 0.806 (95% CI = 0.395–1.647; *p* = 0.555) for “2–5 years”, and 0.553 (95% CI = 0.292–1.047; *p* = 0.069) for “5 years or more.” The *p*-value for heterogeneity across the four duration categories was 0.157 (Wald χ^2^ = 5.209, df = 3) (Supplementary Table 2).

Several sensitivity analyses were conducted to assess the robustness of the findings. When depressive symptoms were defined using the alternative GDS-15-J cutoff of ≥7, the adjusted OR for dark chocolate consumption for >2 years versus rare or no chocolate consumption was 0.629 (95% CI = 0.422–0.938; *p* = 0.023). When the GDS-15-J score was analyzed as a continuous outcome using negative binomial regression, the corresponding RR was 0.894 (95% CI = 0.799–1.000; *p* = 0.050). After excluding antidepressant users, the adjusted OR was 0.725 (95% CI = 0.522–1.005; *p* = 0.054). Modified Poisson regression yielded an adjusted PR of 0.755 (95% CI = 0.566–1.007; *p* = 0.056). Overall, the estimates for the association with dark chocolate consumption for >2 years were generally consistent in direction and magnitude across these analyses (Supplementary Table 3).

Model diagnostics supported the specification of the fully adjusted logistic regression model. Tests based on restricted cubic splines showed no evidence of nonlinearity for age (*p* = 0.904), years of education (*p* = 0.541), BMI (*p* = 0.063), DVS (*p* = 0.774), or MMSE score (*p* = 0.304). All VIFs were <1.5 (maximum, 1.43), and all tolerance values were >0.70. The Hosmer–Lemeshow test yielded χ^2^ = 6.963 (df = 8; *p* = 0.541); the −2 log likelihood was 1987.294, and the Cox–Snell and Nagelkerke R^2^ values were 0.025 and 0.049, respectively. The maximum Cook’s distance was 0.163, and the maximum leverage value was 0.033. In a diagnostic reanalysis excluding the 15 observations with Cook’s distance > 0.10, the adjusted OR for dark chocolate consumption for >2 years was 0.718 (95% CI = 0.515–1.002; *p* = 0.052), similar to the primary estimate.

*Post hoc* sensitivity analyses restricted to participants with higher MMSE scores also yielded estimates in the same direction as the primary analysis. Among participants with MMSE scores ≥ 27 (*n* = 1,802), the adjusted OR for dark chocolate consumption for >2 years was 0.657 (95% CI = 0.431–1.002; *p* = 0.051). Among participants with MMSE scores ≥ 28 (*n* = 1,366), the corresponding adjusted OR was 0.684 (95% CI = 0.417–1.122; *p* = 0.132) (Supplementary Table 4).

The multiple imputation analysis yielded results consistent in direction with those of the complete-case analysis. After multiple imputation of missing BMI and equivalent income values, the pooled adjusted OR for dark chocolate consumption for >2 years versus rare or no chocolate consumption was 0.694 (95% CI = 0.511–0.944; *p* = 0.020). The corresponding estimates were OR = 1.065 (95% CI = 0.814–1.394; *p* = 0.644) for chocolate consumption without dark chocolate and OR = 1.278 (95% CI = 0.886–1.844; *p* = 0.189) for dark chocolate consumption for ≤2 years (Supplementary Table 5).

Variable-specific missing data before the complete-case analysis are summarized in Supplementary Table 6. Among the 3,315 participants evaluated for covariate missingness, equivalent income was missing for 352 (10.6%) and BMI for 9 (0.3%), with both variables missing in 3 participants. Overall, 358 participants had missing data for at least one covariate and were excluded from the complete-case analysis.

Characteristics of participants included in the complete-case analysis and those excluded because of missing analytical data are compared in Supplementary Table 7. The groups differed in several characteristics, including age, sex, years of education, current alcohol consumption, BMI, and MMSE score. Equivalent income was not formally compared between groups because it was missing for 371 (68.1%) of the participants excluded because of missing analytical data.

## 4 Discussion

This study examined the association between dark chocolate consumption and depressive symptoms among community-dwelling older adults in Japan. Among the 2,957 participants included in the complete-case analysis, 727 (24.6%) reported dark chocolate consumption for >2 years, and 273 (9.2%) reported dark chocolate consumption for ≤2 years. In the fully adjusted primary analysis, the OR for depressive symptoms among participants reporting dark chocolate consumption for >2 years was 0.73 (95% CI = 0.53–1.01) compared with those reporting rare or no chocolate consumption. In the additional analysis restricted to chocolate consumers, dark chocolate consumption for >2 years was associated with lower odds of depressive symptoms compared with chocolate consumption without dark chocolate (OR = 0.62, 95% CI = 0.43–0.88). In contrast, the estimates for dark chocolate consumption for ≤2 years were close to the null in both analyses. Furthermore, estimates for the association with dark chocolate consumption for >2 years were generally consistent in direction and magnitude across multiple sensitivity analyses. To the best of our knowledge, this is the first study to examine the association between dark chocolate consumption and depressive symptoms among community-dwelling older adults in Japan. Overall, these findings suggest a cross-sectional association between self-reported dark chocolate consumption for >2 years and lower odds of depressive symptoms, but do not establish a duration-dependent association.

Regarding the association between dark chocolate consumption and depressive symptoms, a cross-sectional study of US adults found that dark chocolate consumers had significantly lower odds of depressive symptoms than non-consumers, whereas no significant association was observed for non-dark chocolate consumers (9). In addition, a randomized controlled trial among menopausal women reported that participants who consumed 12 g per day of dark chocolate for 8 weeks showed significantly lower mean depressive symptom scores compared with those who consumed milk chocolate (34). The results of our study are broadly consistent with those of these previous studies in other adult populations, although differences in study populations, exposure assessment, and study designs should be considered when comparing the findings.

Previous studies differed from the present study in how habitual chocolate consumption was assessed. Jackson et al. assessed chocolate consumption using two 24-h dietary recalls and therefore did not assess consumption duration (9), whereas in the randomized controlled trial by Abdoli et al., the amount and duration of chocolate consumption were specified by the study protocol (34). In contrast, the present study incorporated self-reported frequency and retrospectively reported duration of dark chocolate consumption. This approach allowed us to examine whether consumption duration was related to the observed association with depressive symptoms. However, the lack of clear statistical evidence for heterogeneity across the original duration categories does not support a definitive conclusion that the association differs according to consumption duration. Moreover, because consumption duration was assessed retrospectively in a cross-sectional study, a duration-dependent effect cannot be inferred.

Potential biological mechanisms underlying the observed association may involve polyphenols, particularly flavanols, which are abundant in cocoa. A systematic review of 38 studies by Colombage et al. suggested potential beneficial effects of dietary flavonoids on mood and mental health (35). Cocoa flavanols have antioxidant and anti-inflammatory properties (36), and depressive symptoms have been associated with inflammatory markers such as C-reactive protein and interleukin-6 (37). Flavonoid intake has also been associated with changes in BDNF levels (38), and BDNF is involved in neuroplasticity and has been implicated in depressive symptoms (39). Furthermore, daily consumption of 30 g of 85% cocoa dark chocolate for 3 weeks has been reported to exert prebiotic effects and improve mood through alterations in the gut–brain axis (40). Together, these findings suggest biologically plausible pathways that could potentially contribute to the observed association. However, the present study did not assess cocoa flavanol intake, inflammatory markers, BDNF, or gut microbiota; therefore, these mechanisms remain hypothetical in relation to our findings. Further studies are needed to investigate the biological mechanisms underlying the observed association. From a practical perspective, although the present study assessed the frequency of dark chocolate consumption, it did not assess the amount consumed and was not designed to determine an optimal consumption pattern. Future longitudinal and interventional studies should examine whether particular amounts and frequencies of dark chocolate consumption are prospectively associated with, or result in, clinically meaningful outcomes in older adults, while also considering feasibility, cost, and long-term adherence.

One strength of the present study was that it examined the association between self-reported dark chocolate consumption and depressive symptoms in a relatively large sample of community-dwelling older adults in Japan, with information on both consumption frequency and retrospectively reported duration. However, this study had some limitations. First, because this was a cross-sectional study, a causal association between dark chocolate consumption and depressive symptoms could not be established. The temporal ordering of dark chocolate consumption and depressive symptoms also could not be determined. In addition, reverse causation cannot be ruled out, as depressive symptoms may influence dietary habits. For example, individuals with depressive symptoms may have an altered appetite or reduced motivation for food selection, which could affect the frequency or type of chocolate consumption. Second, the questionnaire items, including those assessing chocolate consumption, were based on self-reported data and may therefore be subject to recall bias and measurement error. In particular, the duration of dark chocolate consumption was assessed retrospectively and required recall over a relatively long period. The chocolate questionnaire used in the present study has not undergone formal validation or reproducibility testing. Furthermore, serving size, grams consumed, number of portions, actual cocoa-polyphenol exposure, exact cocoa percentage, and total energy intake from chocolate were not assessed. Therefore, the exposure assessment in the present study was limited to self-reported frequency and duration of dark chocolate consumption. Additionally, the original consumption-duration categories were grouped into ≤2 years and >2 years for the primary analysis, and this analytical categorization may have influenced the results. Sensitivity analyses restricted to participants with MMSE scores ≥ 27 and ≥28 yielded effect estimates in the same direction as the primary analysis; however, these analyses address only one potential component of recall bias and do not eliminate the possibility of exposure misclassification. Third, although multiple covariates were adjusted for in the analysis, residual and unmeasured confounding cannot be excluded. In particular, individuals reporting dark chocolate consumption for >2 years may differ from the comparison groups in health consciousness, overall dietary patterns, social factors, socioeconomic circumstances, or other health-related characteristics that were not fully captured by the measured covariates. Although the Dietary Variety Score was included to account for one aspect of overall diet, it does not comprehensively capture overall diet quality, total energy intake, or the consumption of individual foods and beverages. Therefore, residual confounding by dietary, social, socioeconomic, and health-related factors may partly explain the observed association. Fourth, the primary analysis was based on complete cases, and participants excluded because of missing analytical data differed from included participants in several characteristics. Therefore, selection bias associated with the complete-case analysis cannot be ruled out. However, multiple imputation of missing BMI and equivalent income values yielded an estimate for dark chocolate consumption for >2 years that was similar in direction and magnitude to that obtained in the complete-case analysis, suggesting that the principal finding was not highly sensitive to the handling of these missing covariate data. Finally, the study participants were community-dwelling older adults aged ≥ 70 years recruited from a single urban area in Japan. Accordingly, the findings should not be generalized to the overall older population in Japan and may not apply to frail or institutionalized older adults, older adults with cognitive impairment, or populations with clinically diagnosed depression.

## 5 Conclusion

Among community-dwelling older adults in Nagoya, Japan, self-reported dark chocolate consumption for >2 years was associated with lower estimated odds of depressive symptoms in the primary and additional analyses, although uncertainty remained in the primary analysis. Sensitivity analyses generally yielded estimates in the same direction. These findings suggest a cross-sectional association between self-reported dark chocolate consumption for >2 years and lower odds of depressive symptoms, but do not establish causality or a duration-dependent association. Future longitudinal and interventional studies are needed to clarify the temporal relationship, assess potentially causal effects, and investigate the underlying biological mechanisms.

## Data Availability

The datasets presented in this article are not readily available as the study participants did not provide consent for their research data to be available to the public without restrictions. Requests to access the datasets should be directed to the corresponding author, SN at: snosaka@ncgg.go.jp.
