# Supplementary material for: Self-reported dark chocolate consumption and depressive symptoms among community-dwelling older adults in Nagoya, Japan: a cross-sectional study
Source: Front Nutr. 2026 Sep 11;13:1924057. doi: 10.3389/fnut.2026.1924057 (PMC13614013; doi:10.3389/fnut.2026.1924057)
Supplement: Supplementary file 1 [file Data_Sheet_1.docx]

STROBE Statement—checklist of items that should be included in reports of observational studies

|  | Item No. | Recommendation | Section heading | Relevant text from manuscript |
| --- | --- | --- | --- | --- |
| **Title and abstract** | 1 | (*a*) Indicate the study’s design with a commonly used term in the title or the abstract | Title | Title: “Self-Reported Dark Chocolate Consumption and Depressive Symptoms among Community-Dwelling Older Adults in Nagoya, Japan: A Cross-Sectional Study.” |
|  |  | (*b*) Provide in the abstract an informative and balanced summary of what was done and what was found | Abstract | Abstract identifies dark chocolate consumption as self-reported and states that dark chocolate consumption and depressive symptoms were assessed at the same study time point. It reports the cross-sectional design, 3,910 participants assessed/2,957 analyzed, exposure and GDS-15-J outcome definitions, adjusted logistic regression results with ORs and 95% CIs, sensitivity analyses, and the conclusion. |
| Introduction | | | |  |
| Background/rationale | 2 | Explain the scientific background and rationale for the investigation being reported | 1 Introduction | Introduction summarizes prior evidence on chocolate/cocoa polyphenols, cognition and psychological well-being, notes limited evidence for depressive symptoms in older adults, and explains the relevance to older Japanese adults. |
| Objectives | 3 | State specific objectives, including any prespecified hypotheses | 1 Introduction | “In the present study, we therefore aimed to examine the association between self-reported dark chocolate consumption and depressive symptoms among community-dwelling older adults in Nagoya, Japan.” |
| Methods | | | |  |
| Study design | 4 | Present key elements of study design early in the paper | 2.1 Design | “This cross-sectional analysis used data from the National Center for Geriatrics and Gerontology–Study of Geriatric Syndromes (NCGG–SGS), a population-based, observational, prospective cohort study.” |
| Setting | 5 | Describe the setting, locations, and relevant dates, including periods of recruitment, exposure, follow-up, and data collection | 2.2 Participants | Residents of Midori Ward, Nagoya City, were recruited from the resident registry; assessments were conducted June 20, 2023–August 22, 2024. The analysis is cross-sectional, so no follow-up period applies to this analysis. |
| Participants | 6 | (*a*) *Cohort study*—Give the eligibility criteria, and the sources and methods of selection of participants. Describe methods of follow-up  *Case-control study*—Give the eligibility criteria, and the sources and methods of case ascertainment and control selection. Give the rationale for the choice of cases and controls  *Cross-sectional study*—Give the eligibility criteria, and the sources and methods of selection of participants | 2.2 Participants | Eligibility: Midori Ward residents aged ≥70 years. A census-based mail invitation was sent to 35,108 residents; 3,910 underwent assessment. Sequential exclusion criteria and the final complete-case sample (n=2,957) are described. |
|  |  | (*b*) *Cohort study*—For matched studies, give matching criteria and number of exposed and unexposed  *Case-control study*—For matched studies, give matching criteria and the number of controls per case | N/A | Not applicable to this cross-sectional study; no matching was used. |
| Variables | 7 | Clearly define all outcomes, exposures, predictors, potential confounders, and effect modifiers. Give diagnostic criteria, if applicable | 2.4 Classification of dark chocolate consumption; 2.5 Definition of depressive symptoms; 2.6 Other covariates | Exposure: four chocolate-consumption groups based on overall/dark-chocolate frequency and duration. Outcome: depressive symptoms defined as GDS-15-J ≥6. Potential confounders include demographic, socioeconomic, lifestyle/dietary, clinical, and MMSE variables. |
| Data sources/ measurement | 8* | For each variable of interest, give sources of data and details of methods of assessment (measurement). Describe comparability of assessment methods if there is more than one group | *2.4 Classification of dark chocolate consumption; 2.5 Definition of depressive symptoms; 2.6 Other covariates* | *Chocolate exposure was assessed by self-administered questionnaire; GDS-15-J assessed depressive symptoms; chronic diseases/antidepressant use were obtained by nurse interview (medication notebooks when needed); BMI, DVS, income, and MMSE assessment methods are described.* |
| Bias | 9 | Describe any efforts to address potential sources of bias | 2.2 Participants; 2.7 Statistical analyses; 4 Discussion | To reduce unreliable questionnaire responses, participants with MMSE <21 were excluded; trained staff used standardized procedures. Sensitivity analyses addressed outcome definition, antidepressant use, cognitive status, missing data, and model influence. Limitations discuss recall/measurement, residual confounding, selection bias, and reverse causation. |
| Study size | 10 | Explain how the study size was arrived at | 2.7 Statistical analyses | “No a priori sample-size calculation was performed specifically for the present analysis because the sample size was determined by the number of eligible participants available from the existing cohort rather than by this specific analysis.” |

Continued on next page

| Quantitative variables | 11 | Explain how quantitative variables were handled in the analyses. If applicable, describe which groupings were chosen and why | 2.4 Classification of dark chocolate consumption; 2.5 Definition of depressive symptoms; 2.6 Other covariates; 2.7 Statistical analyses | Dark-chocolate consumption duration was originally assessed using four questionnaire response categories (“Within 6 months,” “6 months to 2 years,” “2 to 5 years,” and “5 years or more”). These categories were combined into ≤2 years and >2 years for the primary analysis, and the original four categories were examined in a sensitivity analysis. Equivalent income was categorized as <2.0, 2.0–3.99, and ≥4.0 million yen; GDS-15-J was primarily dichotomized at ≥6. |
| --- | --- | --- | --- | --- |
| Statistical methods | 12 | (*a*) Describe all statistical methods, including those used to control for confounding | 2.7 Statistical analyses | Group comparisons used ANOVA/chi-square (Fisher exact for antidepressant use). Logistic regression estimated ORs and 95% CIs; Model 2 adjusted for age, sex, education, income, BMI, smoking, alcohol, DVS, diabetes, hyperlipidemia, hypertension, heart disease, and MMSE. Model diagnostics and two-sided α=0.05 are described. |
|  |  | (*b*) Describe any methods used to examine subgroups and interactions | 2.7 Statistical analyses | Additional analysis was restricted to chocolate consumers using three exposure groups. Post hoc sensitivity analyses were restricted to participants with MMSE ≥27 and ≥28. No interaction analysis is reported. |
|  |  | (*c*) Explain how missing data were addressed | 2.7 Statistical analyses | Primary analysis used complete cases. Multiple imputation by chained equations (50 datasets) was used as a sensitivity analysis for missing BMI and equivalent income; predictive mean matching/polytomous logistic regression and Rubin’s rules were used. |
|  |  | (*d*) *Cohort study*—If applicable, explain how loss to follow-up was addressed  *Case-control study*—If applicable, explain how matching of cases and controls was addressed  *Cross-sectional study*—If applicable, describe analytical methods taking account of sampling strategy | 2.2 Participants; 2.7 Statistical analyses | Participants were recruited using a resident-registry sampling frame with a census-based invitation approach. The statistical analysis section does not report sampling weights or other design-based adjustment. |
|  |  | (*e*) Describe any sensitivity analyses | 2.7 Statistical analyses | Sensitivity analyses examined the original duration categories, GDS-15-J ≥7, the GDS-15-J total score without dichotomization using negative binomial regression, exclusion of antidepressant users, modified Poisson regression, MMSE ≥27/≥28 restrictions, multiple imputation, and influential observations. |
| Results | | | | |
| Participants | 13* | (a) Report numbers of individuals at each stage of study—eg numbers potentially eligible, examined for eligibility, confirmed eligible, included in the study, completing follow-up, and analysed | 2.2 Participants; 3 Results; Figure 1 | 35,108 invited; 3,910 assessed; sequential exclusions are reported; 2,957 included in complete-case analysis. Results also report the numbers in each chocolate-consumption category. |
|  |  | (b) Give reasons for non-participation at each stage | 2.2 Participants; 3 Results; Figure 1 | Reasons for exclusion are reported sequentially: long-term care certification, ADL disability, dementia, MMSE <21, missing exclusion variables, missing exposure/outcome, non-analyzable chocolate responses, and missing covariates. |
|  |  | (c) Consider use of a flow diagram | Figure 1 | Figure 1 is a flowchart of participant selection and classification; the legend states exclusions were sequential and mutually exclusive. |
| Descriptive data | 14* | (a) Give characteristics of study participants (eg demographic, clinical, social) and information on exposures and potential confounders | 3 Results; Table 1 | Participant characteristics are summarized overall and by four chocolate-consumption categories, including demographics, socioeconomic factors, lifestyle/dietary factors, clinical variables, MMSE, and depressive symptoms. |
|  |  | (b) Indicate number of participants with missing data for each variable of interest | 3 Results; Supplementary Table S6 | Variable-specific missing data are reported: equivalent income 352/3,315 (10.6%), BMI 9/3,315 (0.3%), with 358 participants missing ≥1 covariate; exposure/outcome missingness is also tabulated in Supplementary Table S6. |
|  |  | (c) *Cohort study*—Summarise follow-up time (eg, average and total amount) | N/A | Not applicable: the present analysis is cross-sectional and does not report follow-up time. |
| Outcome data | 15* | *Cohort study*—Report numbers of outcome events or summary measures over time | *N/A* | *Not applicable to this cross-sectional study.* |
|  |  | *Case-control study—*Report numbers in each exposure category, or summary measures of exposure | *N/A* | *Not applicable to this cross-sectional study.* |
|  |  | *Cross-sectional study—*Report numbers of outcome events or summary measures | *3 Results; Table 1* | *Depressive symptoms ranged from 8.3% to 13.2% across exposure groups. Table 1 reports depressive-symptom counts and percentages by chocolate-consumption category.* |
| Main results | 16 | (*a*) Give unadjusted estimates and, if applicable, confounder-adjusted estimates and their precision (eg, 95% confidence interval). Make clear which confounders were adjusted for and why they were included | 2.7 Statistical analyses; 3 Results; Tables 2–3 | Crude, age/sex-adjusted, and fully adjusted ORs with 95% CIs and p values are reported. Covariate selection is described as based on demographic, socioeconomic, lifestyle/dietary, and health-related relevance. |
|  |  | (*b*) Report category boundaries when continuous variables were categorized | 2.4 Classification of dark chocolate consumption; 2.5 Definition of depressive symptoms; 2.6 Other covariates | Dark chocolate was defined as ≥70% cocoa; primary duration groups were ≤2 vs >2 years. Equivalent income categories and the GDS-15-J ≥6 outcome threshold are specified; original duration boundaries are also reported. |
|  |  | (*c*) If relevant, consider translating estimates of relative risk into absolute risk for a meaningful time period | N/A | Not applicable: this cross-sectional study reports odds/prevalence estimates rather than risk over a time period. |

Continued on next page

| Other analyses | 17 | Report other analyses done—eg analyses of subgroups and interactions, and sensitivity analyses | 2.7 Statistical analyses; 3 Results; Supplementary Tables S1–S7 | Other analyses included the analysis restricted to chocolate consumers and multiple sensitivity analyses, including the original consumption-duration categories, an alternative GDS-15-J threshold, the GDS-15-J total score without dichotomization, exclusion of antidepressant users, modified Poisson regression, MMSE restrictions, multiple imputation, and model diagnostics. |
| --- | --- | --- | --- | --- |
| Discussion | | | | |
| Key results | 18 | Summarise key results with reference to study objectives | 4 Discussion | The Discussion summarizes the findings relative to the study objective. Dark chocolate consumption for >2 years yielded a lower adjusted OR for depressive symptoms than rare or no chocolate consumption in the primary analysis (OR, 0.73; 95% CI, 0.53–1.01; p=0.055), although uncertainty remained. Among chocolate consumers, dark chocolate consumption for >2 years was associated with lower odds than chocolate consumption without dark chocolate (OR, 0.62; 95% CI, 0.43–0.88; *p*=0.008). |
| Limitations | 19 | Discuss limitations of the study, taking into account sources of potential bias or imprecision. Discuss both direction and magnitude of any potential bias | 4 Discussion | Limitations include the cross-sectional design and possible reverse causation, self-report and recall/measurement error, lack of formal validation of the exposure questionnaire and quantitative intake data, residual/unmeasured confounding, potential selection bias related to complete-case analysis, and limited generalizability. Their potential implications for interpretation are discussed cautiously, although the magnitude of these biases cannot be quantified from the present data. |
| Interpretation | 20 | Give a cautious overall interpretation of results considering objectives, limitations, multiplicity of analyses, results from similar studies, and other relevant evidence | 4 Discussion | Interpretation compares findings with prior observational/intervention studies, discusses possible mechanisms as hypothetical, notes uncertainty regarding duration effects, and emphasizes that cross-sectional data cannot establish temporal or causal relationships. |
| Generalisability | 21 | Discuss the generalisability (external validity) of the study results | 4 Discussion | Generalizability is limited because participants were relatively healthy community-dwelling adults aged ≥70 years recruited from a single urban area in Japan. The findings should not be generalized to the overall older population in Japan and may not apply to frail or institutionalized older adults, older adults with cognitive impairment, populations with clinically diagnosed depression, people in other geographic regions, or populations with different dietary habits. |
| Other information | |  | | |
| Funding | 22 | Give the source of funding and the role of the funders for the present study and, if applicable, for the original study on which the present article is based | 6 Conflict of Interest; 7 Author Contributions; 8 Funding | Funding sources are listed in the Funding section. The relationship with Meiji Co., Ltd. and the roles of Meiji-affiliated coauthors are described in the Conflict of Interest and Author Contributions sections, including their roles in relation to the statistical analyses, interpretation, provision of relevant literature and academic information, manuscript preparation and review, and responsibility for final submission. |

*Give information separately for cases and controls in case-control studies and, if applicable, for exposed and unexposed groups in cohort and cross-sectional studies.

**Note:** An Explanation and Elaboration article discusses each checklist item and gives methodological background and published examples of transparent reporting. The STROBE checklist is best used in conjunction with this article (freely available on the Web sites of PLoS Medicine at http://www.plosmedicine.org/, Annals of Internal Medicine at http://www.annals.org/, and Epidemiology at http://www.epidem.com/). Information on the STROBE Initiative is available at www.strobe-statement.org.
