# Supplementary material for: Self-reported dark chocolate consumption and depressive symptoms among community-dwelling older adults in Nagoya, Japan: a cross-sectional study
Source: Front Nutr. 2026 Sep 11;13:1924057. doi: 10.3389/fnut.2026.1924057 (PMC13614013; doi:10.3389/fnut.2026.1924057)
Supplement: Supplementary file 2 [file Table_1.docx]

Supplementary Material: Chocolate and Cocoa Consumption Questionnaire

| Original Japanese instruction |  |  |  |  |  |
| --- | --- | --- | --- | --- | --- |
| チョコレート・ココアの摂取頻度についておうかがいします。ここ1週間くらいの状況についてお答えください。 | | | | | |
|  |  |  |  |  |  |
| English translation |  |  |  |  |  |
| Please indicate the frequency of your chocolate and cocoa consumption. Please answer based on your consumption during approximately the past week. | | | | | |
|  |  |  |  |  |  |
| Item | Almost every day | Once every 2 days | Once or twice per week | Hardly ever | Unable to answer / Do not know |
| Chocolate overall | □ | □ | □ | □ | □ |
| High-cocoa chocolate (≥70% cocoa) | □ | □ | □ | □ | □ |
| Other chocolate | □ | □ | □ | □ | □ |
| Cocoa (≥1 cup) | □ | □ | □ | □ | □ |
|  |  |  |  |  |  |
| Original Japanese instruction |  |  |  |  |  |
| 高カカオチョコレート・ココアについて、上記の頻度で継続して摂取している期間についておうかがいします。 | | | | | |
|  |  |  |  |  |  |
| English translation |  |  |  |  |  |
| For high-cocoa chocolate and cocoa, please indicate how long you have continuously consumed each at the frequency reported above. | | | | | |
|  |  |  |  |  |  |
| Item | Within 6 months | 6 months to 2 years | 2 to 5 years | 5 years or more | Unable to answer / Do not know |
| High-cocoa chocolate (≥70% cocoa) | □ | □ | □ | □ | □ |
| Cocoa (≥1 cup) | □ | □ | □ | □ | □ |

Note: Frequency and duration of high-cocoa chocolate consumption were assessed separately. Participants who reported consuming high-cocoa chocolate (≥70% cocoa) once or twice per week or more in the frequency question were subsequently asked to report the duration for which they had continuously consumed high-cocoa chocolate at the reported frequency. Participants who reported “hardly ever” for high-cocoa chocolate consumption were not asked the corresponding duration question.

Supplementary Table S1. Cross-tabulation of self-reported dark chocolate consumption frequency and duration among participants consuming dark chocolate at least once or twice weekly

| Dark chocolate consumption frequency | Within 6 months | 6 months to 2 years | 2 to 5 years | 5 years or more | Total |
| --- | --- | --- | --- | --- | --- |
| Once or twice weekly | 70 (10) | 86 (10) | 110 (12) | 309 (22) | 575 (54) |
| Every second day | 19 (2) | 32 (6) | 34 (2) | 94 (6) | 179 (16) |
| Almost daily | 26 (4) | 40 (4) | 63 (7) | 117 (11) | 246 (26) |
| Total | 115 (16) | 158 (20) | 207 (21) | 520 (39) | 1,000 (96) |

Values are presented as number of participants (number of participants with depressive symptoms [GDS-15-J ≥6]). Dark chocolate consumption frequency and duration were self-reported. Consumption frequency referred to the participants’ reported current consumption frequency, whereas consumption duration was assessed retrospectively. Duration categories are presented according to the wording of the original questionnaire response options. Participants who responded “unable to answer/don’t know” to the duration question were excluded from this cross-tabulation because these responses were treated as missing data in the analyses.

Supplementary Table S2. Sensitivity analysis of the association between retrospectively reported duration of dark chocolate consumption and depressive symptoms

| Retrospectively reported duration of  dark chocolate consumption | n | GDS-15-J ≥6, n | Adjusted OR (95% CI) | *p* |
| --- | --- | --- | --- | --- |
| Within 6 months | 115 | 16 | Ref. |  |
| 6 months to 2 years | 158 | 20 | 0.947 (0.458–1.957) | 0.883 |
| 2 to 5 years | 207 | 21 | 0.806 (0.395–1.647) | 0.555 |
| 5 years or more | 520 | 39 | 0.553 (0.292–1.047) | 0.069 |
| *p* for heterogeneity = 0.157 (Wald χ² = 5.209, df = 3) | | |  |  |

The analysis was restricted to 1,000 participants who reported consuming dark chocolate once or twice per week or more frequently and had available data on consumption duration. Values are presented as numbers of participants or adjusted odds ratios (ORs) with 95% confidence intervals (CIs), as appropriate. Dark chocolate consumption frequency and duration were self-reported, and consumption duration was assessed retrospectively. The “Within 6 months” category was used as the reference. Participants who responded “unable to answer/Do not know” to the duration question were excluded because these responses were treated as missing data in the analyses. Duration categories are presented according to the wording of the original questionnaire response options. The model was adjusted for age, sex, years of education, equivalent income, body mass index (BMI), current smoking, current alcohol consumption, Dietary Variety Score (DVS), diabetes mellitus, hyperlipidemia, hypertension, heart disease, and Mini-Mental State Examination (MMSE) score. The *p* value for heterogeneity was calculated using a 3-df Wald test for the original duration categories. Depressive symptoms were defined as a Japanese version of the 15-item Geriatric Depression Scale (GDS-15-J) score ≥6.

Supplementary Table S3. Sensitivity analyses of the association between dark chocolate consumption and depressive symptoms

| Sensitivity analysis | Rare or no  chocolate consumption | Chocolate consumption without dark chocolate | Dark chocolate consumption  for ≤2 years | Dark chocolate consumption  for >2 years |
| --- | --- | --- | --- | --- |
| GDS-15-J ≥7, OR | Ref. | 0.915 (0.645–1.296), *p*=0.616 | 0.942 (0.571–1.552), *p*=0.813 | 0.629 (0.422–0.938), *p*=0.023 |
| Continuous GDS-15-J score, RR | Ref. | 1.031 (0.924–1.150), *p*=0.590 | 0.996 (0.852–1.166), *p*=0.964 | 0.894 (0.799–1.000), *p*=0.050 |
| Excluding antidepressant  users (n=2,938), OR | Ref. | 1.093 (0.817–1.461), *p*=0.550 | 1.168 (0.779–1.751), *p*=0.454 | 0.725 (0.522–1.005), *p*=0.054 |
| Modified Poisson regression, PR | Ref. | 1.087 (0.850–1.390), *p*=0.506 | 1.150 (0.824–1.606), *p*=0.410 | 0.755 (0.566–1.007), *p*=0.056 |

Values are presented as adjusted odds ratios (ORs), rate ratios (RRs), or prevalence ratios (PRs), as appropriate, with 95% confidence intervals (CIs) and *p* values. Rare or no chocolate consumption was used as the reference category. Chocolate consumption categories were based on self-reported consumption, and the duration of dark chocolate consumption was assessed retrospectively. Unless otherwise specified, depressive symptoms were defined as a Japanese version of 15-item Geriatric Depression Scale (GDS-15-J) score ≥6, and ORs were estimated using multivariable logistic regression. For the analysis using a GDS-15-J cutoff of ≥7, depressive symptoms were defined as GDS-15-J ≥7. For the analysis using the continuous GDS-15-J score, RRs and 95% CIs for the expected GDS-15-J score were estimated using negative binomial regression with a log link. For the modified Poisson regression analysis, PRs and 95% CIs were estimated using robust variance estimation. All models were adjusted for age, sex, years of education, equivalent income, body mass index (BMI), current smoking, current alcohol consumption, Dietary Variety Score (DVS), diabetes mellitus, hyperlipidemia, hypertension, heart disease, and Mini-Mental State Examination (MMSE) score. The analysis excluding antidepressant users was restricted to participants who were not using antidepressant medication.

Supplementary Table S4. Sensitivity analyses restricted to participants with higher MMSE scores

| MMSE restriction | Chocolate consumption category | Depressive symptoms,  n (%) | Adjusted OR (95% CI) | *p* |
| --- | --- | --- | --- | --- |
| MMSE ≥27  (n=1,802) | Rare or no chocolate consumption (n=706) | 84 (11.9) | Ref. |  |
|  | Chocolate consumption without dark chocolate (n=456) | 54 (11.8) | 1.034 (0.711–1.503) | 0.861 |
|  | Dark chocolate consumption for ≤2 years (n=164) | 22 (13.4) | 1.133 (0.674–1.903) | 0.638 |
|  | Dark chocolate consumption for >2 years (n=476) | 36 (7.6) | 0.657 (0.431–1.002) | 0.051 |
| MMSE ≥28  (n=1,366) | Rare or no chocolate consumption (n=511) | 58 (11.4) | Ref. |  |
|  | Chocolate consumption without dark chocolate (n=355) | 38 (10.7) | 0.989 (0.633–1.545) | 0.961 |
|  | Dark chocolate consumption for ≤2 years (n=123) | 17 (13.8) | 1.228 (0.673–2.241) | 0.503 |
|  | Dark chocolate consumption for >2 years (n=377) | 27 (7.2) | 0.684 (0.417–1.122) | 0.132 |

Values for depressive symptoms are presented as n (%). Depressive symptoms were defined as a Japanese version of the 15-item Geriatric Depression Scale (GDS-15-J) score ≥6. Adjusted odds ratios (ORs) and 95% confidence intervals (CIs) were estimated using multivariable logistic regression, with rare or no chocolate consumption as the reference category. Chocolate consumption categories were based on self-reported consumption, and the duration of dark chocolate consumption was assessed retrospectively. Models were adjusted for age, sex, years of education, equivalent income, body mass index (BMI), current smoking, current alcohol consumption, Dietary Variety Score (DVS), diabetes mellitus, hyperlipidemia, hypertension, heart disease, and Mini-Mental State Examination (MMSE) score. Analyses were restricted to participants with MMSE scores ≥27 and ≥28, respectively. These analyses were not prespecified and were conducted post hoc to assess the robustness of the findings among participants with relatively preserved cognitive function.

Supplementary Table S5. Multiple imputation analysis of the association between dark chocolate consumption and depressive symptoms

| Variable | Adjusted OR (95% CI) | *p* |
| --- | --- | --- |
| Rare or no chocolate consumption | Ref. |  |
| Chocolate consumption without dark chocolate | 1.065 (0.814–1.394) | 0.644 |
| Dark chocolate consumption for ≤2 years | 1.278 (0.886–1.844) | 0.189 |
| Dark chocolate consumption for >2 years | 0.694 (0.511–0.944) | 0.020 |
| Age, per year | 1.006 (0.984–1.029) | 0.592 |
| Sex (reference: women) | 0.936 (0.722–1.214) | 0.618 |
| Years of education, per year | 0.998 (0.952–1.046) | 0.933 |
| Equivalent income |  |  |
| Low (<2.00 million yen) | Ref. |  |
| Middle (2.00–3.99 million yen) | 0.830 (0.653–1.055) | 0.128 |
| High (≥4.00 million yen) | 0.283 (0.162–0.496) | <0.001 |
| Body mass index, per kg/m² | 0.976 (0.940–1.013) | 0.203 |
| Current smoking (reference: no) | 1.899 (1.211–2.978) | 0.005 |
| Current alcohol consumption (reference: no) | 0.907 (0.715–1.151) | 0.423 |
| Dietary Variety Score, per point | 0.953 (0.904–1.005) | 0.076 |
| Diabetes mellitus (reference: no) | 1.259 (0.940–1.688) | 0.123 |
| Hyperlipidemia (reference: no) | 0.849 (0.668–1.080) | 0.183 |
| Hypertension (reference: no) | 1.048 (0.832–1.320) | 0.691 |
| Heart disease (reference: no) | 1.685 (1.298–2.188) | <0.001 |
| MMSE score, per point | 0.994 (0.947–1.045) | 0.821 |

Values are presented as pooled adjusted odds ratios (ORs) with 95% confidence intervals (CIs). Rare or no chocolate consumption was used as the reference category for the chocolate consumption variable. Chocolate consumption categories were based on self-reported consumption, and the duration of dark chocolate consumption was assessed retrospectively. The model was adjusted for age, sex, years of education, equivalent income, body mass index (BMI), current smoking, current alcohol consumption, Dietary Variety Score (DVS), diabetes mellitus, hyperlipidemia, hypertension, heart disease, and Mini-Mental State Examination (MMSE) score. Depressive symptoms were defined as the Japanese version of the 15-item Geriatric Depression Scale (GDS-15-J) score ≥6. Missing values in BMI and equivalent income were imputed using multiple imputation by chained equations with 50 imputed datasets; predictive mean matching was used for BMI and polytomous logistic regression for equivalent income. Estimates were pooled across the imputed datasets using Rubin’s rules.

Supplementary Table S6. Variable-specific missing data before complete-case analysis

| Variable | Role | Missing, n | Missing, % |
| --- | --- | --- | --- |
| Exposure and outcome (n = 3,502) |  |  |  |
| Chocolate consumption status (4-category variable) | Exposure | 7 | 0.2 |
| GDS-15-J score | Outcome | 1 | <0.1 |
| Covariates (n = 3,315) |  |  |  |
| Age | Covariate | 0 | 0.0 |
| Sex | Covariate | 0 | 0.0 |
| Years of education | Covariate | 0 | 0.0 |
| Equivalent income | Covariate | 352 | 10.6 |
| Body mass index | Covariate | 9 | 0.3 |
| Current smoking | Covariate | 0 | 0.0 |
| Current alcohol consumption | Covariate | 0 | 0.0 |
| Dietary Variety Score | Covariate | 0 | 0.0 |
| Diabetes mellitus | Covariate | 0 | 0.0 |
| Hyperlipidemia | Covariate | 0 | 0.0 |
| Hypertension | Covariate | 0 | 0.0 |
| Heart disease | Covariate | 0 | 0.0 |
| MMSE score | Covariate | 0 | 0.0 |

Values are presented as numbers and percentages of participants with variable-specific missing data. Missingness of the exposure and outcome was assessed among 3,502 participants remaining after sequential application of the eligibility and exclusion criteria. After excluding 8 participants with missing exposure or outcome data and 179 participants with non-analyzable responses to the chocolate-consumption questions, covariate missingness was assessed among 3,315 participants. Missingness across covariates was not mutually exclusive; therefore, the numbers of participants with missing covariate data should not be summed across variables. Of the 3,315 participants, 352 had missing data on equivalent income and 9 had missing BMI data, including 3 participants with missing data for both variables; thus, 358 participants had missing data for at least one covariate. After excluding these participants, 2,957 participants were included in the complete-case analysis. GDS-15-J, Japanese version of the 15-item Geriatric Depression Scale; BMI, body mass index; MMSE, Mini-Mental State Examination.

Supplementary Table S7. Comparison of characteristics between participants included in the complete-case analysis and those excluded because of missing analytical data or non-analyzable responses

| Characteristic | Excluded because of missing analytical data or non-analyzable responses  (n=545) | Included in  complete-case  analysis (n=2,957) | *p* value |
| --- | --- | --- | --- |
| Age, years | 79.46 ± 5.17 | 77.96 ± 5.02 | <0.001 |
| Women, n (%) | 324 (59.4) | 1,432 (48.4) | <0.001 |
| Years of education, years | 12.43 ± 2.51 | 13.08 ± 2.55 | <0.001 |
| Equivalent income, n (%) |  |  |  |
| Low (<2.00 million yen) | 79 (14.5) | 1,310 (44.3) |  |
| Middle (2.00–3.99 million yen) | 69 (12.7) | 1,303 (44.1) |  |
| High (≥4.00 million yen) | 26 (4.8) | 344 (11.6) |  |
| Body mass index, kg/m² | 22.53 ± 3.21 | 22.91 ± 3.10 | 0.009 |
| Current smoking, n (%) | 17 (3.1) | 142 (4.8) | 0.083 |
| Current alcohol consumption, n (%) | 200 (36.7) | 1,346 (45.5) | <0.001 |
| Dietary Variety Score, points | 5.01 ± 2.25 | 4.86 ± 2.17 | 0.134 |
| Diabetes mellitus, n (%) | 84 (15.4) | 449 (15.2) | 0.891 |
| Hyperlipidemia, n (%) | 203 (37.2) | 1,094 (37.0) | 0.911 |
| Hypertension, n (%) | 272 (49.9) | 1,478 (50.0) | 0.974 |
| Heart disease, n (%) | 113 (20.7) | 556 (18.8) | 0.292 |
| MMSE score, points | 26.38 ± 2.45 | 26.84 ± 2.29 | <0.001 |

Values are presented as mean ± standard deviation or number (percentage), as appropriate. Participants excluded because of missing analytical data comprised those with missing data on chocolate consumption or depressive symptoms, those with non-analyzable responses to the chocolate-consumption questions, and those with missing covariate data. Continuous variables were compared using independent-samples t tests; Welch’s t test was used when the assumption of equal variances was not met. Categorical variables were compared using Pearson’s chi-square tests. Data were based on available observations for each variable; therefore, denominators may vary because of missing data. Equivalent income was missing for 371 (68.1%) participants in the excluded group and was therefore not formally compared between groups. BMI, body mass index; MMSE, Mini-Mental State Examination.
